# Splenic Lymphoma Mimicking a Hydatid Cyst Causing a Diagnostic Challenge: A Case Report

**DOI:** 10.7759/cureus.73077

**Published:** 2024-11-05

**Authors:** Kenana Altell, Wasef Alhroub, Maaweya Jabareen, Ammar Hassouneh

**Affiliations:** 1 Department of Surgery, College of Medicine, Hebron University, Hebron, PSE; 2 Department of Radiology, Hebron Governmental Hospital, Hebron, PSE

**Keywords:** diffuse large b-cell lymphoma, gi malignancy, hydatid cyst, primary splenic dlbcl, spleen

## Abstract

Splenic lymphoma, particularly primary splenic diffuse large B-cell lymphoma (PS-DLBCL), is a rare malignancy that often presents with nonspecific symptoms, complicating diagnosis. This case report describes a 44-year-old female with left flank pain and nausea whose imaging studies, including ultrasound and CT, revealed a well-defined heterogeneous lesion in the spleen and left pleural effusion, initially suggesting a hydatid cyst. Despite negative serological tests for echinococcosis, clinical suspicion remained due to the endemic presence of the disease. Owing to atypical imaging features and the patient’s worsening condition, a splenectomy was performed, revealing diffuse large B-cell lymphoma upon histopathological examination.

## Introduction

Splenic lymphoma is one of the rarest hematological malignancies that affects the spleen, accounting for a small percentage of primary extranodal lymphomas [1]. Splenic lymphoma, like the rest of the hematological malignancies, presents with non-specific and vague symptoms such as abdominal pain, and B symptoms, which are night sweats, unintentional weight loss, and fever [2].

Imaging studies, such as ultrasound or CT scans, often reveal splenic lesions that may resemble other more common splenic pathologies, complicating the diagnosis [3]. One of the clinical cases that may mimic splenic lymphoma is a hydatid cyst, which is a parasitic infection caused by *Echinococcus granulosus* that may affect the spleen as well, especially in endemic areas like the Mediterranean region, Africa, and South America [4].

Differentiating between splenic lymphoma and splenic hydatid cysts is crucial due to the vastly different treatment approaches required. While splenic lymphoma typically necessitates chemotherapy or immunotherapy, hydatid cysts are treated with antiparasitic drugs like Albendazole or surgical excision [5]. Misdiagnosis could result in unnecessary surgical intervention or delays in initiating life-saving cancer therapy.

The prognosis for splenic lymphoma tends to differ significantly from other splenic disorders. For example, splenic lymphomas generally require aggressive treatment but have varied outcomes based on subtype, while benign splenic conditions typically have a more favorable prognosis. This difference highlights the importance of accurate diagnosis and tailored management.

This study aims to illustrate the diagnostic challenge of distinguishing splenic lymphoma from hydatid cysts by presenting a case where splenic lymphoma was initially misdiagnosed as a hydatid cyst. Through this case, we highlight the importance of a comprehensive diagnostic approach, including advanced imaging techniques and histopathological confirmation, to avoid diagnostic errors in similar clinical scenarios.

## Case presentation

A 44-year-old female presented to the emergency department with a one-day history of intermittent left-sided flank pain radiating to her left shoulder. She also experienced nausea, difficulty breathing, unexplained weight loss, and night sweats but reported no accompanying symptoms like fever.

During the physical examination, the patient exhibited signs of discomfort; however, the abdomen was soft and lax, with tenderness noted in the left flank and epigastric regions. The spleen was non-palpable, with no signs of abdominal distention, lymphadenopathy, or organomegaly. Chest auscultation revealed decreased air entry on the left side.

**Figure 1:**
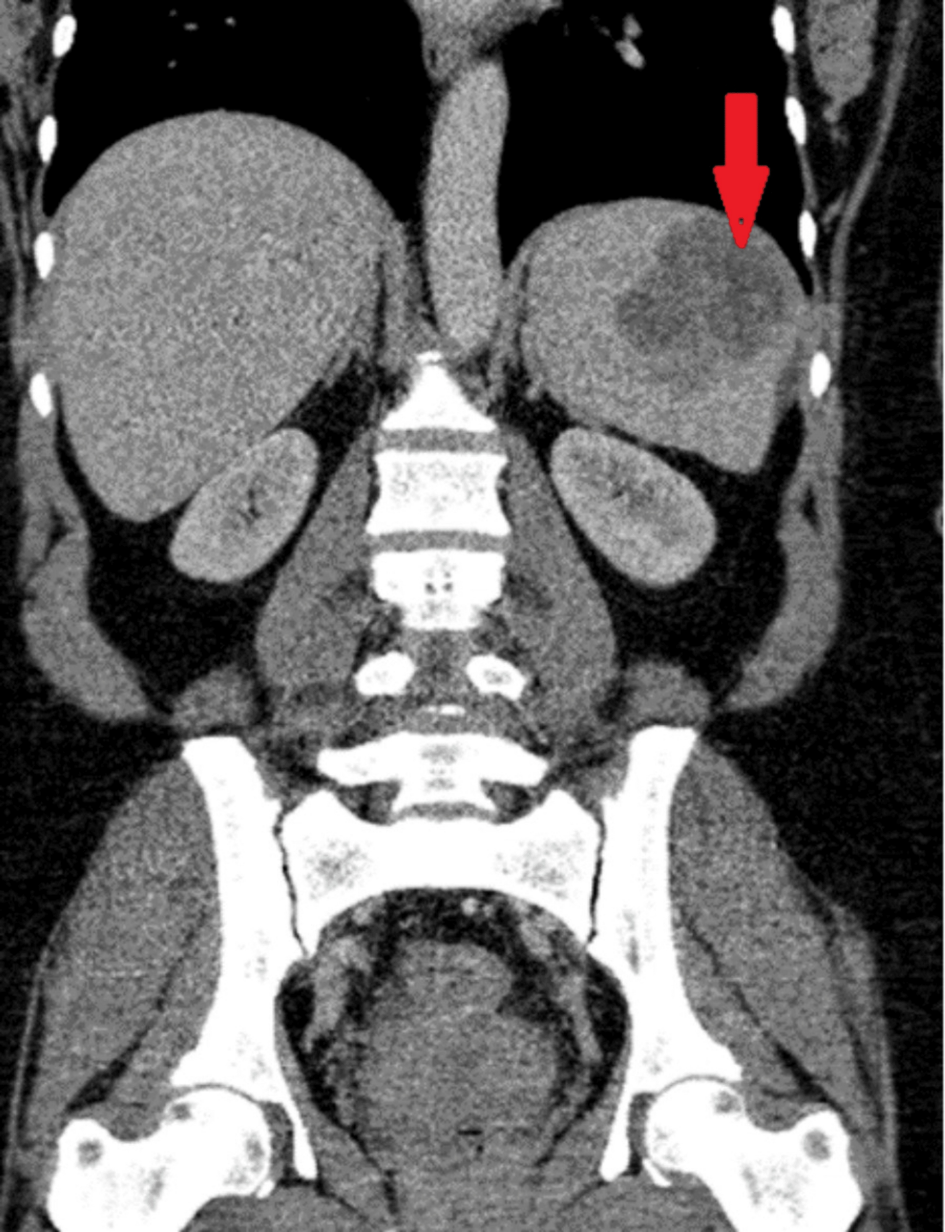
CT abdomen with IV contrast-portal phase, in coronal plane, showed splenic, subcapsular, heterogenous predominantly hypodense lesion with internal streak enhancing components (red arrow).

**Figure 2:**
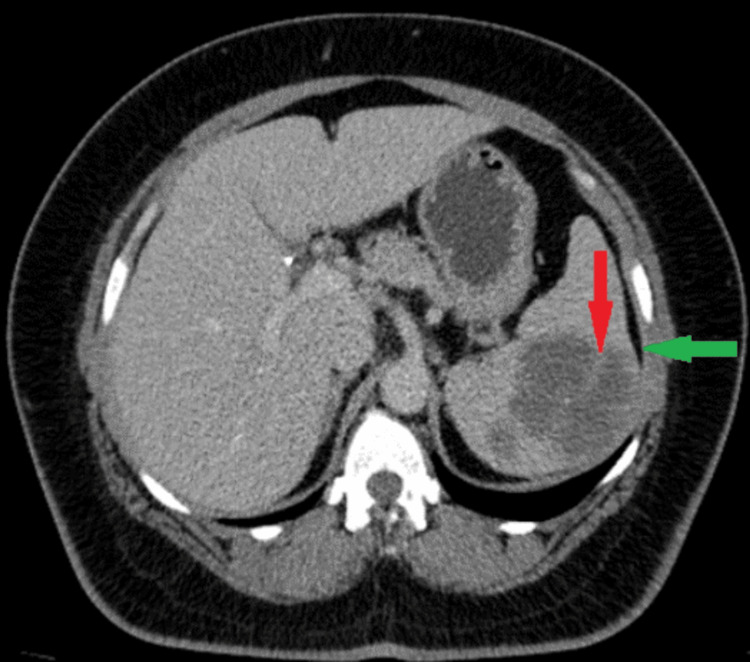
CT abdomen with IV contrast-portal phase, in axial plane, showed splenic, subcapsular, heterogenous predominantly hypodense lesion with internal streak enhancing components (red arrow), irregular contour of the lesion, and mild bulging of capsule was noted (green arrow).

Abdominal ultrasound revealed mild splenomegaly, with the spleen measuring approximately 14.5 cm. Additionally, a well-defined, heterogeneous area was noted in the upper pole of the spleen, measuring around 5 x 3.5 cm. A computed tomography (CT) scan was performed for the chest and the abdomen and showed a splenic, subcapsular, heterogeneous, predominantly hypodense lesion with internal streak enhancing components, the irregular contour of the lesion, and mild bulging of the capsule (Figures 1, 2). A complete blood count with differential showed some abnormal results, including an elevated white blood cell count. Additionally, the patient was found to have anemia, as seen in Table 1.

**Table 1:** Complete blood count.

Lab	Results	Reference
Hemoglobin	10.4 g/dl	11.1-14.5 g/dL
Hematocrit	30.4%	35.4%-42.0%
RBC count	3.82 × 10^6/µL	3.9-5.5 × 10^6/µL
Mean corpuscular volume	79.6 fL	76.0-96.0 fL
Mean corpuscular hemoglobin	27.2 pg	26.0-32.0 pg
Mean corpuscular hemoglobin concentration	34.2 g/dL	32.0-36.0 g/dL
WBC count	21.4 × 10^9/L	4.0-10.0 × 10^9/L
Neutrophils granulocytes	19.3	-
Lymphocytes	7.4	-
Monocytes	2.3%	2%-10%
Platelets	539 × 10^9/L	150-400 × 10^9/L
K+	4.6 mmol/L	3.5-5.0 mmol/L
Glucose	90 mg/dL	65-99.0 mg/dL
Ca^2+^	10.2 mg/dL	8.7-10.2 mg/dL
Blood urea nitrogen	25 mg/dL	6-20 mg/dL
Creatinine	1.69 mg/dL	0.76-1.27 mg/dL
Alanine aminotransferase	50 UI/L	0-40 UI/L
Aspartate aminotransferase	50 UI/L	0-40 UI/L
Serum lactate dehydrogenase	309 U/L	207-414 U/L
Uric acid	7.30 mg/dl	3.7-7.1 mg/dl

**Figure 3:**
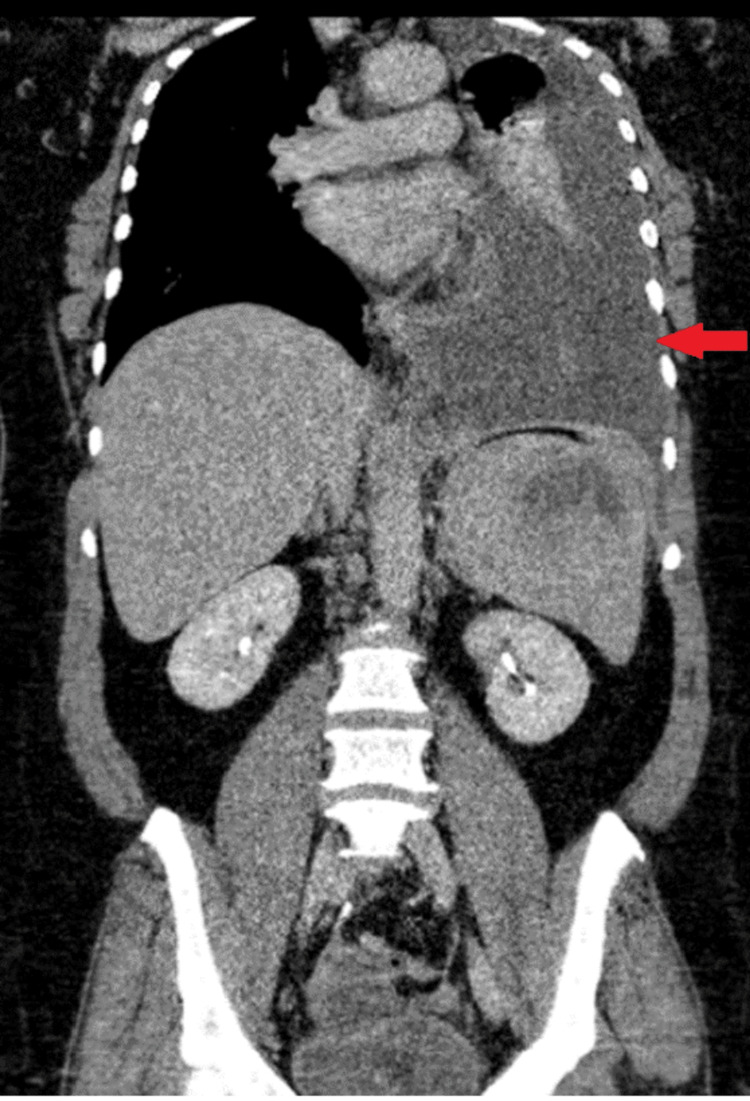
Follow-up CT abdomen with IV contrast in the coronal view after three months showed rapid development of large left-sided pleural effusion (red arrow) with passive collapse consolidation of left lung lobes, with no signs of mediastinal lymph nodes.

**Figure 4:**
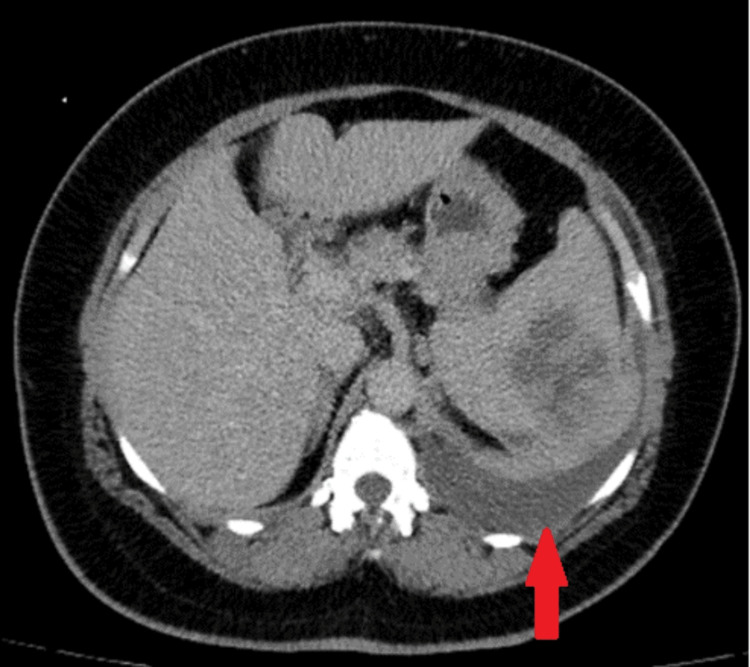
Follow-up CT abdomen with IV contrast in the axial view after three months showed rapid development of large left-sided pleural effusion (red arrow) with passive collapse consolidation of left lung lobes, with no signs of mediastinal lymph nodes.

A diagnosis of splenic hydatid cyst was established, leading to a three-month course of Albendazole and the administration of appropriate vaccinations. However, she returned to the emergency room with a rapid decline in abdominal condition and severe dyspnea. A follow-up CT abdomen with IV contrast showed rapid development of a large left-sided pleural effusion with passive collapse and consolidation of the left lung lobes, with no signs of mediastinal lymph node involvement, as shown in Figures 3, 4. Differential diagnosis was discussed between splenic ruptured hydatid cyst and splenic lymphoma with malignant pleural effusion. A splenectomy was carried out, and histopathological analysis of the lesion confirmed diffuse large B-cell lymphoma (DLBCL). The patient has begun chemotherapy, with a full recovery anticipated in six months. Follow-up over the subsequent six months revealed normal laboratory results with no signs of recurrence.

## Discussion

The case presented revealed the diagnostic dilemma posed by the clinical and radiologic overlap between splenic lymphoma and splenic hydatid cysts. Splenic involvement by lymphoma is rare, representing about 1% of all lymphomas, but when it occurs, it can mimic other cystic or solid lesions in the spleen, such as hydatid cysts, particularly in endemic areas [6]. In this case, the initial misdiagnosis of a hydatid cyst delayed the correct diagnosis of splenic lymphoma, underscoring the importance of careful evaluation when assessing splenic lesions.

DLBCL is one of the most common non-Hodgkin lymphomas, with a higher prevalence in older adults and more male predominance. While the spleen can be involved, DLBCL is often seen in lymph nodes, gastrointestinal tract, and bone marrow. Uncommonly, extranodal sites such as the skin or central nervous system may be affected, so the prognosis varies significantly based on this and on factors such as age and performance status. The overall survival rates have improved with advancements in treatment, yet early and accurate diagnosis remains crucial for better outcomes [7].

In the context of splenic lymphoma, particularly DLBCL, histopathological findings often reveal sheets of atypical lymphoid cells with high mitotic activity, indicating aggressive behavior. These neoplastic cells may infiltrate the splenic tissue, leading to splenomegaly, which can sometimes be misinterpreted as a hydatid cyst. Clinically, patients may present with symptoms such as abdominal pain, weight loss, fever, and night sweats, which can overlap with those of hydatid disease, making diagnosis challenging. Management typically involves a combination of chemotherapy, particularly with regimens like rituximab, cyclophosphamide, doxorubicin, vincristine, and prednisone (R-CHOP) and, in some cases, splenectomy [8].

Hydatid disease is an endemic parasitic infection in many regions caused by *Echinococcus granulosus*, including parts of the Mediterranean, the Middle East, and South America. Splenic hydatid cysts are relatively rare, accounting for less than 5% of all abdominal hydatid disease cases [9]. In regions where hydatid disease is prevalent, splenic cystic lesions are often assumed to be hydatid cysts, as was seen in this case, leading to a potential misdiagnosis, so it must always be considered in the differential diagnosis list.

However, there are some key differences in the radiologic findings of these two conditions that, when carefully evaluated by experts, can aid in differentiation between them. Hydatid cysts typically present as well-defined cystic lesions with characteristic features like calcifications or the presence of daughter cysts on imaging [9]. In contrast, splenic lymphoma, although it can present as a cystic lesion, often exhibits more heterogeneity, including solid components on imaging, as our patient had [10].

Despite these distinctions, both conditions can present with nonspecific clinical symptoms such as abdominal discomfort, pain, and splenomegaly [11]. In this case, a more thorough imaging assessment with advanced techniques, such as contrast-enhanced CT or MRI, might have revealed features more suggestive of lymphoma rather than a hydatid cyst. Moreover, early biopsy or fine-needle aspiration of the lesion could have led to an earlier definitive diagnosis as well [12].

The diagnostic workup for splenic masses typically begins with an ultrasound, followed by contrast-enhanced CT or MRI to further characterize any suspicious lesions. When lymphoma is suspected, positron emission tomography (PET)/CT is often recommended, as it helps identify the hypermetabolic activity indicative of malignancy. Laboratory findings, such as elevated lactate dehydrogenase (LDH) levels, can also support suspicion of lymphoma. Prognosis for splenic lymphoma varies depending on the subtype; some cases require aggressive treatment with regular monitoring through imaging and blood tests. Follow-up protocols generally involve periodic CT scans to detect recurrence or progression, along with ongoing symptom assessment. To prevent similar diagnostic errors in the future, it is crucial to follow a structured diagnostic algorithm and engage a multidisciplinary team for complex cases. Careful assessment of imaging findings in conjunction with clinical data can help avoid misdiagnosis, and retrospectively reviewing cases with an honest appraisal of potential oversights can improve diagnostic accuracy [12].

The delayed diagnosis in this case also emphasizes the need for a multidisciplinary approach in the evaluation of splenic lesions, particularly in endemic areas. Collaborating with infectious disease specialists, radiologists, hematologists, and pathologists can help ensure that all differential diagnoses, including malignancies like lymphoma, are considered before proceeding with treatment. Misdiagnosis of splenic lymphoma as a hydatid cyst can lead to unnecessary surgical intervention or delayed chemotherapy, both of which can significantly impact patient outcomes.

Finally, this case highlights the importance of considering splenic lymphoma in the differential diagnosis of cystic splenic lesions, even in regions where hydatid disease is common. As evidenced by this case, thorough diagnostic evaluation remains essential to avoid delays in appropriate treatment and to improve prognosis.

## Conclusions

This case highlights the significant diagnostic challenges posed by splenic lymphoma that can mimic a hydatid cyst. The overlapping clinical and radiological features between these two conditions necessitate a comprehensive and multidisciplinary diagnostic approach, particularly in endemic regions where hydatid disease is prevalent. Given the potential for misdiagnosis, it is essential to prioritize early biopsy when diagnostic uncertainty remains after imaging, as it can confirm malignancy sooner, allowing for timely intervention and potentially improving patient outcomes. In cases where lymphoma is suspected but cannot be confirmed through imaging alone, biopsy should be prioritized. Delayed diagnosis of splenic lymphoma can have significant implications for patient outcomes, often resulting in disease progression to more advanced stages that require intensive treatment and may carry a poorer prognosis. Timely diagnosis is crucial for implementing an effective treatment plan and minimizing complications. Follow-up for splenic lymphoma should include periodic CT or PET/CT scans to monitor for recurrence or progression, along with regular clinical evaluations and laboratory testing (such as LDH) to detect early changes. In high-risk cases, closer follow-up intervals are recommended, especially in the initial years post-treatment. Ultimately, this approach is crucial in improving patient outcomes and minimizing the risks associated with delayed treatment, reinforcing the importance of vigilance and thoroughness in clinical practice.
